# Erratum: NeuroDecodeR: a package for neural decoding in R

**DOI:** 10.3389/fninf.2024.1408064

**Published:** 2024-04-16

**Authors:** Frontiers Production Office

**Affiliations:** Frontiers Media SA, Lausanne, Switzerland

**Keywords:** neural decoding, readout, multivariate pattern analysis, R, data analysis, statistics, machine learning, data science

Due to a production error, the code phrase “cl_max_correlation” was incorrectly written as “cl:max_correlation” on four occasions in the online webpage version of this article.

A correction has been made to the section **Decoding analysis 1: decoding face identity using left profile images**, subsection **Running the decoding analysis**, paragraph 2:

“3. cl_max_correlation classifier to make our predictions.”

A correction has been made to the section **Decoding analysis 1: decoding face identity using left profile images**, subsection **Running the decoding analysis**, paragraph 3:

“ds < - ds_basic(binned_data=“FV_AM_30bins_10

sampled.Rda”,

labels= “orient_person_combo”,

num_cv_splits=3,

label_levels=left_profile_levels,

site_IDs_to_use=sites_to_use)

fps < - list(fp_zscore())

cl < - cl_max_correlation()

rms < - list(rm_main_results(), rm_confusion_matrix())

cv < - cv_standard(datasource=ds, classifier=cl, feature_preprocessors=fps, result_ metrics=rms)”

A correction has been made to the section **Decoding analysis 2: testing generalization across head orientations**, paragraph 5:

“fps < - list(fp_zscore())

cl < - cl_max_correlation()

rms < - list(rm_main_results())

cv < - cv_standard(datasource=ds,

classifier=cl,

feature_preprocessors=fps,

result_metrics=rms,

run_TCD=FALSE)

DECODING_RESULTS < - run_decoding(cv)

log_save_results(DECODING_RESULTS,

save_directory_name= “results”,

result_name= “Train left profile, test

right profile”)”

A correction has been made to the section **Decoding analysis 2: testing generalization across head orientations**, subsection **Piping together NeuroDecodeR objects**, paragraph 2:

““FV_AM_30bins_10sampled.Rda” |>

ds_basic(labels= “orient_person_combo”,

num_cv_splits=3,

label_levels=right_profile_levels,

site_IDs_to_use=sites_to_use) |>

fp_zscore() |>

cl_max_correlation() |>

rm_main_results() |>

rm_confusion_matrix() |>

cv_standard(run_TCD=FALSE) |>

run_decoding() |>

log_save_results(save_directory_

name= “results”,

result_name=“Right profile face

decoding”)

result_names < - c(result_names, “Right profile

face decoding”)

plot_main_results(results_dir_name= “results”,

result_names)”

The publisher apologizes for this mistake. The original article has been updated.

