# Supplementary material for: NeuroDecodeR: a package for neural decoding in R
Source: Front Neuroinform. 2024 Jan 3;17:1275903. doi: 10.3389/fninf.2023.1275903 (PMC10791947; doi:10.3389/fninf.2023.1275903)
Supplement: Supplementary file 1 [file Data_Sheet_1.docx]

Supplementary Material

NeuroDecodeR: A package for neural decoding in R

Ethan M. Meyers

*** Correspondence:** Ethan M. Meyers: [ethan.meyers@yale.edu](mailto:ethan.meyers@yale.edu)

**Supplemental Figure**


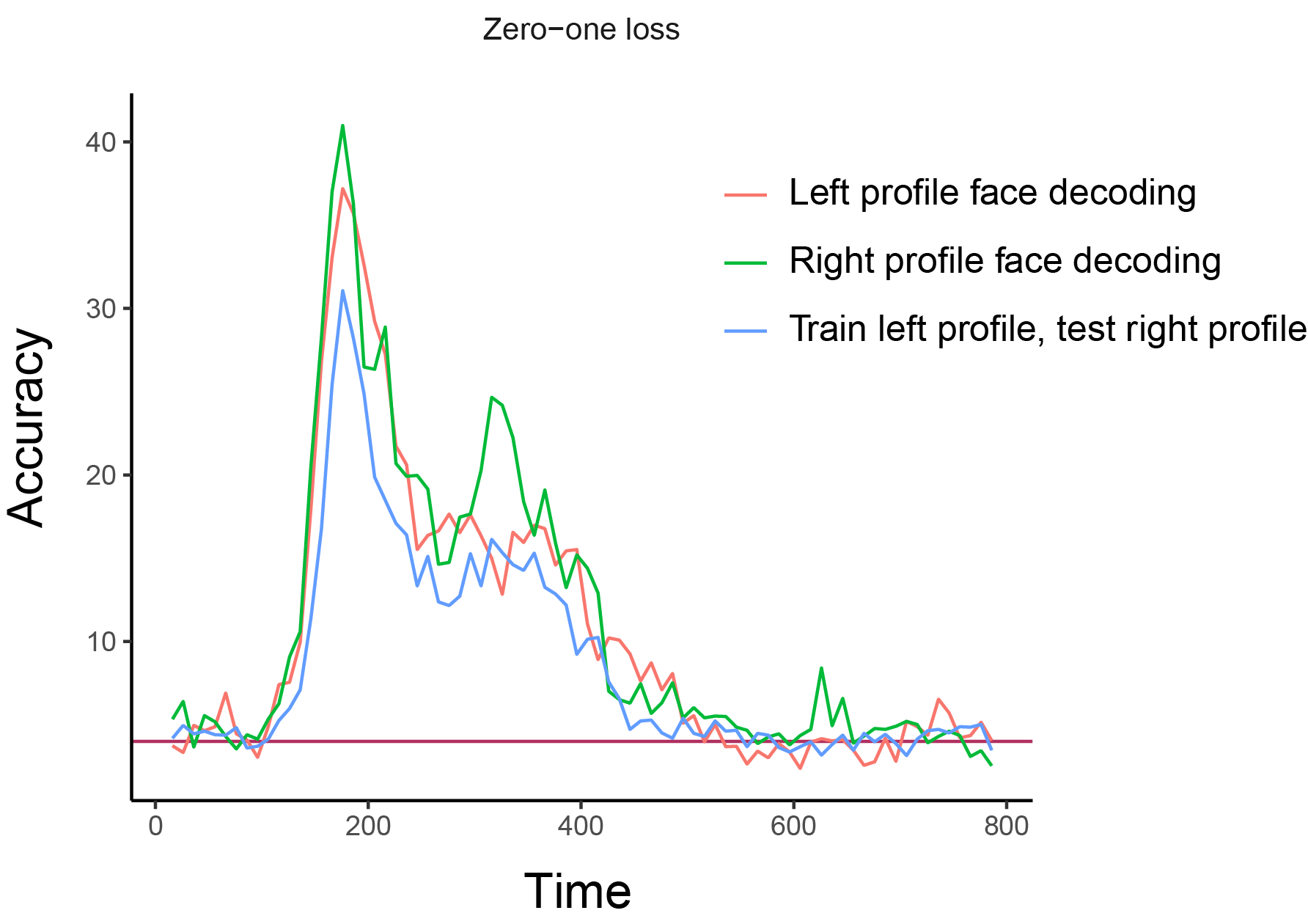


**Supplemental figure 1.** **Comparison of basic identity decoding results for left and right profile images, to generalization analysis results.** The plot shows the results from classifying the 25 individual face identities using a basic decoding analysis where the classifier was trained and tested using on left profile face images (red trace), or on right profile images (green trace) to generalization analysis results where the classifier was trained on left profile images and tested on right profile images (blue trace); i.e., these are the same results as figure 5 except the basic decoding results of training and testing on the right profile image have been added to the plot. Again, we see the classification accuracies are fairly similar for the basic and generalization analysis indicating that face identity information in brain region AM is contained in a code that is highly invariance to the pose of the head across left and right profile images. The x-axis shows the time from stimulus onset in milliseconds, and the y-axis is the classification accuracy (0-1 loss).

Datasources (ds):

- **ds_basic:** creates basic training and test splits of the data, including the ability to create pseudo-populations.
- **ds_generalization**: extends the ds_basic classifier and allows one to run a generalization analysis where the training set consists of one set of experimental conditions (as indicated by the training label levels) and the test set consists of a different related set of conditions (as indicated by the test label levels).

Feature-preprocessors (fp):

- **fp_zscore**: calculates the mean and standard deviation of each feature on the training set, and then z-score normalizing the training and test set features using these values. This is useful if particular features tend to have larger values than others (e.g., if there are some neurons that have higher firing rates than others).
- **fp_select_k_features**: finds the k most selective features on the training data, and then eliminates all other features from the training and test set. This is useful to assess whether there is a small subset of features that contains all the information that is available.

Classifiers (cl):

- **cl_max_correlation**: A maximum correlation coefficient classifier. This classifier uses the training data to learn one prototype (average) vector for each class. It then calculates the correlation between a test point and each of these prototype vectors and returns the class with the highest correlation as its prediction, and the correlation values as its decision values.
- **cl_poisson_naive_bayes**: A Poisson Naïve Bayes classifier. This classifier uses the training data to learn rate parameters of the Poisson distributions for class and each feature. It then makes predictions by calculating the log-likelihood that a test point belongs to each class and returns the class with the highest likelihood as its prediction and the log-likelihood values of the decision values. Note: to use this classifier, the data needs to be integer values (e.g., spike counts).
- **cl_svm**: A support vector machine classifier. This classifier uses the e1071 package to implement a Support Vector Machine. To do multiclass classification, an all-pairs classification scheme is used, where a binary classifier is trained to discriminate between all pairs of classes. A test point is then assigned to the class that was predicted most often by these binary classifiers, and the decision values are the number of contests won by each class.

Result metrics (rm):

- **rm_main_results**: Calculates three measures of decoding accuracy which are:
  - 1. The classification accuracy (zone-one loss), which is the percent of trials correctly classified.
  - 2. Normalized rank results, which is a number indicating how far down on an ordered list of predictions the correct prediction is. A value of 1 means the first prediction was the correct prediction, while a value of 0.5 means the correct prediction is halfway down the ordered list of predictions and indicates a chance level of performance.
  - 3. Decision values which are the average decision values returned by the classifier.

Plotting functions for this object include the ability to create line plots showing the different measures of decoding accuracy as a function of time, and temporal cross-decoding plots showing how well training at one time period generalizes to making predictions at a different time period.

- **rm_confusion_matrix**: Creates a confusion matrix showing how often trials from class *i* were predicted to belong to class *j*. The associated plot function allows one to see a sequence of confusion matrices as a function of time.

Cross-validators (cv):

- - **cv_standard**: This runs a cross-validation loop where: 1) training and test splits of data are pulled from a datasource; 2) this data is passed to any feature pre-processors that have been specified to do pre-processing of the data; 3) the data is then pass to a classifier which builds a predictive model on the training data and then makes predictions on the test data; 4) the predictions are then passed to the result metric objects which create aggregate measures of decoding accuracy; 5) runs steps 1-4 in several ‘resample runs’ (which can be run in parallel), and the final aggregated decoding accuracies are returned in the result metric objects.

**Supplemental Table 1. More detailed description of implementations of NeuroDecodeR abstract objects that come with the NeuroDecodeR package.** The package also includes a number of additional functions that are useful for plotting and processing the data, and for saving and loading results. For more details on these objects, see the online documentation at: <https://emeyers.github.io/NeuroDecodeR/reference/index.html>

**Methods all NeuroDecodeR objects need to implement**

- **contructor:** All NeuroDecodeR objects need to have a constructor that can return an instance of an object.
- **get_parameters:** This function returns a data frame that has a single row and a column for each parameters that can be set during the construction of the object. This function is used by the log_save_results() function to keep a record of which parameters were used in an analysis.

**Datasources (DS)**

- **get_data():** This function returns a training and test set of data that is used by a FP and CL objects.

**Feature preprocessors (FP)**

- **preprocess_data():** This function takes training and test data. It learns any relevant parameters from the training data and returns modified training and test data sets after preprocessing has been applied.

**Classifiers (CL)**

- **get_predictions():** This function takes training and test data, learns a model on the training data, and then returns the predicted class (and optionally decision values) for each point in the test set.

**Result Metrics (RM)**

- **aggregate_cv_split_results():** Takes predictions made over many cross-validation splits and consolidates the predictions into a measure of decoding accuracy.
- **aggregate_resample_run_results():** Takes measures of decoding accuracy that were generated over multiple resample runs (i.e., as returned by earlier calls to the aggregate_cv_split_results() function) and aggregates the results into an overall classification accuracy.

**Cross validators (CV)**

- **run_decoding():** Takes DS, FP, CL and RM objects and runs a full cross-validation procedure returning RM objects that contain different measures of decoding accuracy.

**A list of functions that each NeuroDecodeR needs to implement.** Above is a list of methods that each NeuroDecodeR object needs to implement to work with other objects in the NeuroDecodeR package; i.e, when extending the NeuroDecodeR package with a new DS, FP, CL, RM, or CV NeuroDecodeR object, one needs to implement the methods listed above to create a valid instance of a particular object type. More information about how to implement. new NeuroDecodeR objects, and the data types that these objects operate on, is on the NeuroDecodeR documentation page at: <https://emeyers.github.io/NeuroDecodeR/articles/NDR_object_specification.html>

# The constructor just returns an empty list with the class attribute set to "cl_min_angle"

cl_min_angle <- function() {

the_classifier <- list()

attr(the_classifier, "class") <- "cl_min_angle"

the_classifier

}

# Implements of the get_predictions() method which takes training and test data, and returns predicted labels from the test set

get_predictions.cl_min_angle <- function(cl_obj,training_set, test_set) {

### Train the classifier ---------------------------------------------------

# create the prototypes by taking the mean of feature vectors from each class

prototypes <- training_set |>

dplyr::group_by(train_labels) |>

dplyr::summarise_all(mean)

# separate the labels and data

prototype_labels <- prototypes$train_labels

prototypes <- as.matrix(prototypes[, 2:ncol(prototypes)])

prototypes <- prototypes/sqrt(rowSums(prototypes * prototypes))

### Test the classifier ---------------------------------------------------

test_data <- as.matrix(dplyr::select(test_set, -"test_labels", -"time_bin"))

test_data <- test_data/sqrt(rowSums(test_data * test_data))

# calculate the cosine of the angle between the prototypes and test data

prototype_test_angle <- prototypes %*% t(test_data)

# get the predicted labels as the maximum cosine value

predicted_inds <- apply(prototype_test_angle, 2, which.max)

predicted_labels <- prototype_labels[predicted_inds]

# return a data frame that has all the results

data.frame(test_time = test_set$time_bin, actual_labels = test_set$test_labels, predicted_labels = predicted_labels)

}

# Have the get_parameters() method return a data frame saying that this classifier has no settable parameters

get_parameters.cl_min_angle <- function(ndr_obj) {

data.frame(cl_min_angle.cl_min_angle = "does not have settable parameters")

**}**

**An example of extending the NeuroDecodeR package by creating a new classifier.** The code above illustrates how one can implement a new classifier (a minimum angle classifier) to extend the functional of the NeuroDecodeR package. In particular, to add a new classifier to the package, one needs to implement the get_predictions() method, which takes training and test data, learns a model on the training data and then makes predictions on the test data. One should also implement a construction to create the classifier, and as well as a get_parameters() that returns information about any free parameters the classifier has. Information about how to extend other types of objects can be found at: <https://emeyers.github.io/NeuroDecodeR/articles/NDR_object_specification.html>
